# Oral health community engagement programs for rural communities: A scoping review

**DOI:** 10.1371/journal.pone.0297546

**Published:** 2024-02-06

**Authors:** Hlulani Alloy Nghayo, Celeste Ellouise Palanyandi, Khabiso Jemima Ramphoma, Ronel Maart

**Affiliations:** 1 Faculty of Dentistry, Department of Community Oral Health, University of the Western Cape, Cape Town, South Africa; 2 Faculty of Science, Department of Sport, Rehabilitation and Dental Sciences, Tshwane University of Technology, Pretoria, South Africa; 3 Faculty of Dentistry, Department of Prosthodontics, University of the Western Cape, Cape Town, South Africa; Alexandria University Faculty of Dentistry / Ludwig Maximillians Universitat, EGYPT

## Abstract

This scoping review aims to identify the available literature on oral health community engagement programs that have been developed to guide oral health care in rural communities and to summarize their outcomes. This review was conducted using the 5-stage scoping review framework outlined by Arksey and O’Malley. We conducted a literature search with defined eligibility criteria through electronic databases such as Science Direct, PubMed, ProQuest, Scopus, EBSCOhost, and Wiley Online; other well-established online scientific health and dental organizations such as the WHO, the Fédération Dentaire Internationale of the World Dental Federation, the American Dental Association, and the South African Dental Association; and grey literature spanning the time interval from January 2012 to August 2023. The charted data were classified, analysed, and reported using descriptive and thematic analyses. A total of 19 records were included in the final review. These records were classified into four categories of interventions: community-based, school-based, integrated dental-based, and non-dental volunteer oral health programs. The findings imply that there is a growing appreciation for the significance of qualitative data in enhancing oral healthcare interventions and outcomes. Furthermore, the study showed that oral health strategies were successful in shaping the understanding and perception of oral health among children and mothers/caregivers, and in improving the oral health and quality of life of edentulous older adults and children living in rural communities.

## Introduction

The Global Burden of Disease (GBD) study has shown that the prevalence of common oral diseases remains a significant global health issue [1], affecting an estimated 3.5 billion people worldwide and having a major impact on health, well-being, health care systems, and economies, as well as the increasing burden of Non-Communicable Diseases [2]. Moreover, the global burden of untreated oral diseases, the continued absence of universal health coverage, the cost of basic oral health care for significant portions of the global population, and the escalation of disparities indicate that oral health has not been regarded as a public health priority [3,4].

In developing countries, access to suitable oral healthcare interventions is commonly absent [5]. As a result, disadvantaged communities are still disproportionately affected by oral diseases and are more likely to face barriers in accessing and utilizing oral health care services [6]. Despite significant progress in the prevention and treatment of oral diseases, as well as overall improvements in oral health in recent years, disparities persist, and there is a definite common discrepancy in oral health that reflects that of general health. [7]. This has resulted in significant disparities in global oral health.

Although developing countries are confronted with prevalent challenges such as lack of oral health awareness, limited access to professional dental care services, inadequate transportation options, perceived lack of need for dental care, and obstacles associated with language and culture [8–10], a lack of dental professionals is the primary cause of severely reduced accessibility to oral health services and poor oral health status, not only in developing countries but worldwide [4,11–13].

To address this, the World Health Organization has launched oral healthcare programs, particularly for disadvantaged countries, which include oral health education and the integration of health education with other oral health practices, such as preventive, restorative, and emergency dental care. These programs aim to enhance oral health services within member countries with a particular focus on the most disadvantaged communities [5]. The goal is to ensure equal access to information and resources for high-quality oral health care, provide specialized knowledge for executing clinical trials, and create cost-effective alternatives to increase the availability of oral health services [14].

Several global studies have shown that implementing community-based initiatives for oral health promotion can improve community engagement, leading to the development and improvement of knowledge, attitudes, and behaviours related to oral health. These initiatives have also proven effective in involving communities in promoting long-term oral health [15–18]. Furthermore, the active and meaningful engagement of communities and civil society are essential components of any comprehensive strategy or initiative aimed at achieving oral health objectives and targets included in the Sustainable Development Goals (SDG 3 –good health and well-being, SDG 4 –quality education, SDG 10 –reduced inequality, and SDG 17 –partnerships to achieve the goal) [19].

Implementing mandatory community-based programs for oral health promotion and prevention is crucial. Early detection and treatment of oral diseases can prevent their progression and improve overall health. It is imperative to develop initiatives that cater to underprivileged communities. These initiatives have the potential to contribute significantly to achieving Sustainable Development Goals and ensuring Universal Health Coverage for all. Therefore, this scoping review aims to identify oral health community engagement programs that guide oral health care in rural communities and to provide an overview of their outcomes.

## Materials and methods

This study used the methodological framework for scoping reviews defined by Arksey and O’Malley [20]. The aim of conducting a scoping review is to comprehensively examine and identify the fundamental ideas and themes related to a particular research subject along with the primary sources and various forms of evidence that exist. The scoping review was guided by five stages: identification of the research question, identification of relevant studies, study selection, data charting, and collating, summarizing, and reporting the results. The sixth stage was optional and was excluded upon consensus among the four reviewers (HAN, CEP, KJR, and RM).

### Identification of the research question

The process of conducting this scoping review was guided by a specific research question that informed the selection of relevant literature. The research question formulated for this scoping review was as follows: *What are the oral health community engagement programs that guide oral health care in rural communities*?

### Identification of relevant studies

To identify appropriate studies, Arksey and O’Malley argued that it is necessary to define a search plan based on the location, type, or parameters of the study [20]. The Preferred Reporting Items for Systematic Reviews and Meta-Analyses Extension for Scoping Reviews (PRISMA-ScR) [21] were used to conduct a comprehensive literature review. Using Boolean operators, the key terms and Medical Subject Headings were combined, and an example of a search string (S1 Table) constructed and used in this manner was as follows:—(Oral OR dental) AND (health) AND (rural OR remote) AND (communities OR settings OR areas) AND (engagement OR participation OR outreach OR programs) AND (programs OR programmes OR strategies OR initiatives). The following databases were used to search for all relevant and published journal articles: Science Direct, PubMed, ProQuest, Scopus, EBSCOhost, and Wiley Online. Google Scholar was also used to maximise the search. A health science librarian was consulted to guide the search strategies. Furthermore, Google Scholar and other well-established online scientific health and dental organizations such as the WHO, the Fédération Dentaire Internationale of the World Dental Federation (FDI), the American Dental Association (ADA), and the South African Dental Association (SADA) were used to search for grey literature.

### Study selection

Two independent reviewers (HAN, and CEP) screened the titles and abstracts of each article and identified articles for full review. EndNote reference manager was used to eliminate duplicate articles. Any uncertainties and disagreements were discussed and resolved by consensus.

### Inclusion criteria

Peer-reviewed journal articles published in English between January 2012 and August 2023 were included as part of the inclusion criteria for this scoping review. Articles referring to Oral Health Community Engagement Programs for Rural Communities were eligible for inclusion. In addition, established online scientific health and dental organizations, including ADA, FDI, SADA, and WHO, were searched for grey literature, and academic dissertations were also considered.

### Exclusion criteria

Non-English and non-peer-reviewed journal articles published before 2012 were excluded. In addition, editorials, commentaries, and reviews were excluded, along with all articles that did not reference Oral Health Community Engagement Programs for Rural Communities.

### Data charting

One reviewer (HAN) charted all the data from the included articles based on (a) Bibliographic details: *Author (s)*, *Year*, *Country*; (b) Study aim; (c) Study design; (d) Participant demographics of rural communities; (e) Type of oral health community engagement programs; (f) Duration of oral health community engagement programs/strategies (g) Outcome measures and (h) Key Findings/results. Thereafter, the other reviewer (CEP) extensively analysed the extracted data to validate its accuracy. Any disagreements and rectifications were agreed upon by consensus.

### Collating, summarizing, and reporting the results

Based on the charted data, two reviewers (HAN and CEP) developed categories and subcategories for programs related to oral health community engagement in rural communities. The other two reviewers (KJR and RM) reviewed and verified the categories to ensure consistency and authenticity. The charted data were then grouped, analysed, and reported through descriptive and thematic analyses.

### Ethical considerations

This scoping review formed part of the research project, which was approved by the Biomedical Science Research Ethics Committee of the University of the Western Cape (BMREC) (reference number: BM23/6/16). Informed consent was not obtained for this study because no participants were involved.

## Results

### Characteristics of the included publications

**Fig 1:**
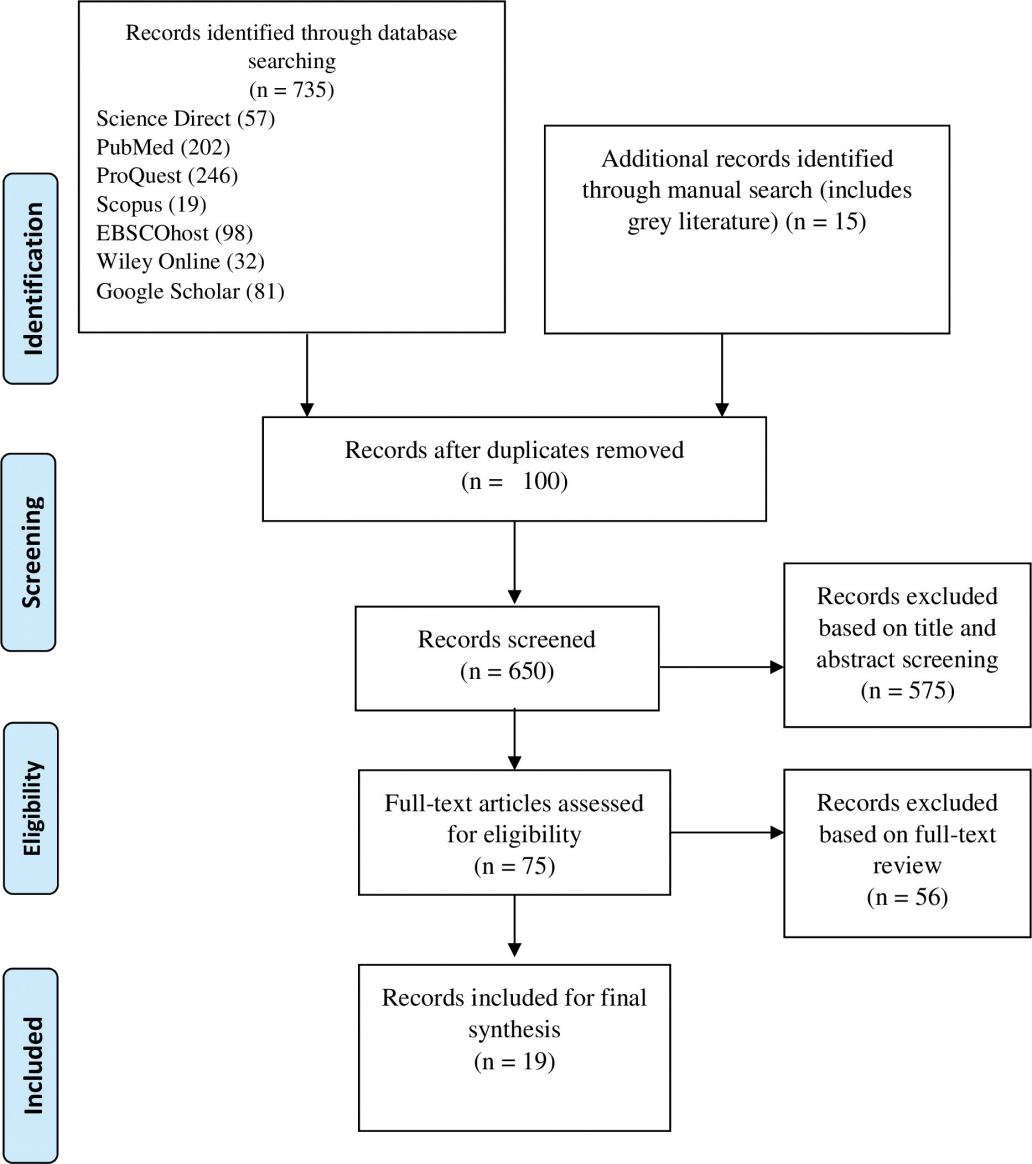
PRISMA flow diagram for study.

Of the 750 articles found through online databases and manual searches, 100 duplicates were eliminated. Of the remaining 650 articles, 575 were excluded due to irrelevance. A total of 75 articles were screened and 19 were selected for full-text review. These 19 articles were later deemed eligible for the scoping review based on the established inclusion criteria **(Fig 1)**. The published research articles identified and included in this scoping review are summarized in **Table 1**.

**Table 1:** Summary of the final articles included in the scoping review.

Author, Year, Country	Aim of the study	Type of study	Study design	Participant demographics	Type of oral health programs/strategies	Outcome measures	Key Findings/Results
Åstrøm (2012) [18]	To assess the validity of the TPB components in predicting changes in intended and self-reported oral health-related behaviours focusing on school-going adolescents from a deprived district of Tanzania.	Original research	Pre-Post Interventional study	1306 pupils in 6^th^ year in primary public school	School-based atraumatic restorative treatment (ART) /oral health education (OHE) program	Improvement of tooth brushing, knowledge, and attitudes among pupils	• Attitudes towards sugar avoidance, knowledge, and tooth brushing improved. • Tooth brushing increased in students with improved oral knowledge.
Chandrashekar *et al*. (2014) [22]	To compare the oral hygiene, plaque, gingival, and dental caries status among rural children receiving dental health education by qualified dentists and schoolteachers with and without supply of oral hygiene aids.	Original research	Pre-Post Interventional study	141 children aged 15 years from four schools	School-based Teacher-Dentist Dental Health Education (DHE) program	Reduction in the oral hygiene index-simplified, plaque index, and gingival index	• Pre- and post-intervention comparison showed a significant decrease in the mean oral hygiene index-simplified (OHI-S), plaque index (PI), and gingival index (GI)
Damle *et al*. (2014) [17]	To evaluate and compare the effectiveness of supervised toothbrushing and its impact on oral health in school children of urban and rural communities.	Original research	Comparative study	197 schoolchildren in the 12–15 age group from two schools	School-based Toothbrushing Teaching Program	Effective establishment of good oral health habits among school children	• Plaque and gingival score reduction was higher in the study groups compared to the control groups. • Increased DMFT and DMFS scores in children throughout the study period
Fernando *et al*. (2015) [16]	To test if we could improve the oral health of preschool children by educating mothers/caregivers.	Original research	Quasi-experimental study	219 mother/child pairs of permanent residents	Community-based Non-Dental Personnel Program	Improvement of the oral health of preschool children	• Dental caries prevalence decreased after six months. • The need for treatment and preventive care decreased after the intervention.
Schroth *et al*. (2015) [15]	To evaluate the impact of a project based on community-development principles to improve community and caregiver knowledge and awareness, and improve ECOH in four distinct Manitoba communities.	Original research	Cross-sectional study	319 children and their primary caregivers	Community-developed Early Childhood Oral Health Collaborative Project (program)	Improvement in caregiver knowledge, attitudes, and behaviours toward early childhood oral health	• Improvements in caregiver knowledge and attitudes • No significant change in early childhood caries prevalence between follow-up and baseline investigations.
Dabiri *et al*. (2016) [23]	To evaluate the impact of oral health education and fluoride in reducing ECC and mouth pain in those communities.	Scientific research report	Retrospective analysis study	Document review	Community-based Early Childhood Caries Intervention Program	Improvement in children’s oral health and quality of life	• Community oral health education and fluoride supplementation programs reduced caries experience
Huang *et al*. (2020) [24]	To explore the effectiveness of an oral hygiene program combined with home-phone health-promoting counselling for rural adults with metabolic syndrome.	Original research	Prospective quasi-experimental	136 community adults living in two rural townships	Community-based Oral Hygiene Program	Improvement of awareness and adoption of a healthy lifestyle	• The intervention group exhibited a greater improvement in the use of dental floss and regular tooth scaling health-promoting behaviours than the control group.
Skinner *et al*. (2020) [25]	To investigate the feasibility of using Aboriginal dental assistants to provide regular fluoride varnish applications for Aboriginal children in the primary school setting.	Quality improvement report	Mixed-methods approach	8 Aboriginal dental assistants	Fluoride Varnish Program Aboriginal Dental Assistant Scholarship Program.	Dental Assistants can safely apply fluoride varnish	• Aboriginal dental assistants can safely apply fluoride varnish in primary schools with remote supervision.
Narayan *et al*. (2021) [26]	To compare and evaluate the changes in OHRQoL after insertion of complete dentures among beneficiaries of the denture programs, as well as compare the responsiveness (longitudinal validity) of OHIP-14 and GOHAI.	Original research	Prospective pre-post comparison study	123 edentulous senior citizens >60 years	Public-Funded Oral Rehabilitation Program (Public-funded denture program)	Effectiveness in the improvement of oral health quality of life (OHRQoL) among the edentulous elderly	• Prosthetic dental rehabilitation provides psychological, social, and functional benefits to the edentulous elderly. • Public‑funded denture programs are effective in improving OHRQoL among the edentulous elderly from poor socioeconomic backgrounds.
Shinde *et al*. (2023) [27]	To implement and evaluate community participatory oral health promotion and prevention programs in schoolchildren from select rural populations in Deshmukhwadi, Pune district, and also carry out knowledge, attitude, and practice surveys among health workers and schoolteachers about oral health.		Pilot Interventional study	139 schoolchildren aged 6–13 years	School-based Participatory Oral Health Promotion and Prevention Program	Improvement in the knowledge and practice of health workers and the filled and initial lesion surfaces in the primary and permanent dentition of school children	• Significant improvement in gingival status, caries status, and incipient lesion post-program • Improvement in the knowledge, attitude, and practice regarding oral health among health workers and schoolteachers post-program
Patel *et al*. (2015) [28]	To determine the potential role volunteers play in improving oral health outcomes for Australian Aboriginal communities.	Original research	Cross-sectional study	42 remote area dental volunteers	Volunteer-based dental organization–Kimberly Dental Team program	Enabling good oral health in remote Aboriginal communities.	• Access to reliable and culturally appropriate care was recognised as a key enabler of good oral health, along with education. • Lack of access to services, poor nutrition, and limited government support were identified as obstacles.
Caldwell *et al*. (2017) [29]	To understand whether a usual source of health care helps mitigate racial differences in complete tooth loss and a recent dental visit among urban and rural older adults.	Original research	Cross-sectional study	15,473 adults aged 50 years and older	Primary Care for Oral Health	Improvement of oral health outcomes, but it did not close the racial gap	• Access to primary health care was associated with improved oral health outcomes
Erchick *et al*. (2020) [30]	To evaluate the validity of periodontal examinations conducted by auxiliary nurse midwives in a rural home setting in Nepal.	Original research	Prospective cohort study	21 pregnant women < 26 weeks gestation	Auxiliary care community-based program	Potential shift in the delivery of certain basic oral health services from dentists and other highly trained professionals to auxiliary nurse midwives	• Auxiliary nurse midwives tend to report higher periodontal probing depth scores compared to dentists.
Sajid *et al*. (2020) [31]	To investigate the role of oral health education programs in oral health status in people living in rural areas of Multan	Original research	Cross-Sectional Survey	380 patients visiting the hospital in Jahangirabad	Community-based Oral Hygiene Program and Adult Health Promotion Program	Improvement in the knowledge and perception of oral health.	• Observed association between higher education and better oral health index. • No significant association found between income and oral health status.
Skinner *et al*. (2021) [32]	To determine the level of satisfaction of graduates and host site supervisors with the Dalang Project and whether the program expanded local oral health service delivery capacity and employment in rural and regional areas of NSW	Original research	Online survey.	15 graduates	Oral Health Therapy Graduate Year Program Integrated into Dalang Project	Oral health therapists remaining in rural, remote, and regional locations	• Program provided regular dental health education to schools, preschools, and community health services. • Program distributed toothbrushes and fluoride toothpastes to children and families.
Akera *et al*. (2022) [33]	To explore teacher contributions to oral health promotion in an existing school program following the WHO health-promoting school framework.	Original research	Qualitative descriptive study	18 primary school teachers	WHO health-promoting school framework.	Implementation of key principles of the WHO’s health-promoting school framework	• Demonstrating oral hygiene care and proper tooth brushing through training. • Raising health awareness with oral health information using various educational methods. • Conducting oral health examinations on children, providing first aid, referring for dental treatments, and involving parents, students, and health workers in oral health promotion.
Patel *et al*. (2022) [34]	To investigate the perceptions and attitudes of oral health among Aboriginal Australians living in remote Kimberley communities in the context of better understanding existing and informing future prevention and education strategies.	Original research	Qualitative descriptive study	80 Aboriginal adults	Community-based Yarning Program	Improvement in oral health outcomes through school-based oral health promotion	• School-based oral health promotion and community-driven restrictions on sugary food and drink sales were deemed effective for oral health improvement. • Modern technology, the internet, and local stores impacted remote communities by introducing new challenges and changing priorities.
Pawloski *et al*. (2022) [35]	To elicit perspectives and experiences of providers and administrators involved in the MDI program to assess the acceptability, feasibility, and success of an MDI integration strategy in Eastern Washington.	Original research	Descriptive qualitative study	12 medical and dental providers and clinical administrators	Medical–Dental Integration (MDI) Program	Identification of facilitators associated with integrating medical and dental care	• MDI program is feasible and acceptable. • Program benefits clinics, and patients, and increases access to quality care.
Ward *et al*. (2022) [36]	To describe how school-based teledentistry programs increased access to oral health services for children and adolescents living in rural areas based on results from two grantee organizations.	Original research	Prospective observational cohort design	1,467 school children aged 3 to 5 years from 57 CDS school sites 164 school children aged 3 to 5 years from 7 MCHS school sites	School-Based Telehealth Network Grant Program	Increase in access to needed oral health care services for rural children	• Increased access to needed oral health care services for rural children. • Provision of an efficient approach to identify students at high risk for dental caries and offer a valuable strategy for oral disease prevention and control.

The majority (n = 11) of the 19 studies included in this scoping review were conducted in developing countries, while only (n = 8) were conducted in developed countries **(Table 2)**. That is, four studies were conducted in Australia and India, respectively, while three were conducted in the USA. Canada, El Salvador, Nepal, Pakistan, Sri Lanka, Taiwan, Tanzania, and Uganda each produced one. The definition of rural community is complicated and has multiple connotations, such as farms, ranches, villages, small towns, open spaces, and low population density, which researchers and policymakers have used inconsistently [37]. However, for this scoping review, rural communities have been defined as disadvantaged and vulnerable settings owing to their low socioeconomic status, limited access to transportation, scarce availability of quality oral health care, insufficient number of oral health care providers, and limited access to oral health education, all of which led to high rates of non-communicable diseases and poor general health [38,39].

**Table 2:** Place of origin of studies.

Study	Developed country	Developing country	Region	Classification
Åstrøm (2012) [18]		**x**	Tanzania **(East Africa)**	Rural
Chandrashekar *et al*. (2014) [22]		**x**	India **(South Asia)**	Rural
Damle *et al*. (2014) [17]		**x**	India **(South Asia)**	Rural
Fernando *et al*. (2015) [16]		**x**	Sri Lanka **(South Asia)**	Rural
Schroth *et al*. (2015) [15]	**x**		Canada **(North America)**	Rural
Dabiri *et al*. (2016) [23]		**x**	El Salvador **(Central America)**	Rural
Huang *et al*. (2020) [24]		**x**	Taiwan **(East Asia)**	Rural
Skinner *et al*. (2020) [25]	**x**		Australia **(Oceania)**	Rural
Narayan *et al*. (2021) [26]		**x**	India **(South Asia)**	Rural
Shinde *et al*. (2023) [27]		**x**	India **(South Asia)**	Rural
Patel *et al*. (2015) [28]	**x**		Australia **(Oceania)**	Rural
Caldwell *et al*. (2017) [29]	**x**		USA **(North America)**	Rural
Erchick *et al*. (2020) [30]		**x**	Nepal **(South Asia)**	Rural
Sajid *et al*. (2020) [31]		**x**	Pakistan **(South Asia)**	Rural
Skinner *et al*. (2021) [32]	**x**		Australia **(Oceania)**	Rural
Akera *et al*. (2022) [33]		**x**	Uganda **(East Africa)**	Rural
Patel *et al*. (2022) [34]	**x**		Australia **(Oceania)**	Rural
Pawloski *et al*. (2022) [35]	**x**		USA **(North America)**	Rural
Ward *et al*. (2022) [36]	**x**		USA **(North America)**	Rural

The majority (n = 13) of the studies employed a quantitative research approach, while (n = 4) of the studies were qualitative, and only (n = 2) employed a mixed-method approach. Most of the included articles sampled primary school children (n = 6), whereas (n = 4) of the articles used community members (adults), dental personnel, and non-dental personnel as study participants. Overall, (n = 1) of the studies used community members (patients) as study targets.

### Identification of oral health community engagement programs

Based on the findings of this scoping review, oral health community engagement programs were grouped into four distinct intervention categories that have been successfully implemented in diverse rural communities. The initial category elucidates the community-based dental interventions [15,22–27]. The subsequent category delineates the school-based dental interventions implemented in the primary school setting [17,18,28–30]. The third category emphasizes the importance of integrated dental-based interventions [25,28,32,35]. The fourth category characterises the auxiliary care community-based interventions [16,35,36]. The summary of categories of oral health community engagement interventions and their programs are outlined and depicted in **Table 3**.

**Table 3:** Summary of categories of oral health community engagement interventions and programs.

Oral Health Community Engagement interventions	Programs
Community-based	• Early Childhood Oral Health Collaborative Project program [15]• Early Childhood Caries Intervention Program [23]• Oral Hygiene Program [24]• Public‑Funded Oral Rehabilitation Program (Public‑funded denture program) [26]• Primary Care for Oral Health [29]• Oral Health Education Program [31]• Yarning Program [34]
School-based	• Atraumatic Restorative Treatment (ART)/Oral Health Education (OHE) Program [18] • Dental Health Education (DHE) program [22] • Toothbrushing Teaching Program [17] • Participatory Oral Health Promotion and Prevention Program [27] • Telehealth Network Grant Program [36]
Integrated dental-based	• Aboriginal Dental Assistant Scholarship Program + Fluoride Varnish Program [25] • Volunteer-based dental organization–Kimberly Dental Team [28] • Oral Health Therapy Graduate Year Program Integrated into Dalang Project [32] • Medical–Dental Integration (MDI) Program [35]
Non-dental volunteer	• Non-Dental Personnel Program [16] • Auxiliary care community-based program [30] • WHO health-promoting school framework [33]

### Category 1: Community-based oral health community engagement interventions

This category encompasses articles on community-based oral health programs implemented to guide oral health care through the adoption of participatory community-based approaches. These articles mainly involve the integration of community members into oral health programs while also addressing social and environmental factors that act as substantial barriers to accessing optimal oral health. The two studies included in this scoping review were conducted in rural communities in Canada and El Salvador and aimed to enhance community and caregiver knowledge and awareness, improve early childhood oral health [6], and assess the impact of oral health education and fluoride on reducing early childhood caries [8] in a 5-year community-based early childhood oral health intervention program. Both studies concluded that the program was successful in improving caregivers’ understanding of and attitudes toward early childhood oral health, and it led to a significant reduction in early childhood caries.

In an ongoing oral health education program, Sajid *et al*. (2020) [13] found a significant association between higher education and better oral health status for dental caries and periodontal disease in a rural Pakistani community. In addition, improvement in knowledge and perception of oral health was observed among individuals within the community. However, the authors concluded that community-related factors, including family, teachers, and dental health personnel of school-based programs, have a positive impact on oral health and should be integrated into oral health education programs to enhance the knowledge and perception of oral health.

Similarly, Australian remote Aboriginal adults were engaged in an ongoing community-based Yarning Program where their perceptions and attitudes towards oral health were investigated to better understand existing prevention and education strategies and inform future ones. Consequently, remote Aboriginal adults indicated that existing school-based oral health promotion and community-driven initiatives have put restrictions on the sale of sugary food and drinks, and these initiatives were seen as positive strategies for improving oral health. However, the availability of the Internet and fixed community stores were perceived as creating new challenges and shifting priorities for those living in remote communities [18].

Huang *et al*. (2020) conducted a study in a rural community in Taiwan, in which 136 community-dwelling adults with metabolic syndrome were recruited from two rural townships. These individuals were invited to participate in an 18-month community-based oral hygiene program. This study aimed to investigate the efficacy of a comprehensive oral hygiene intervention coupled with telephone health counselling among individuals residing in rural communities who were diagnosed with metabolic syndrome. The program demonstrated efficacy in enhancing awareness and mitigating Cardiometabolic risks, along with notable improvements in oral hygiene and health-related behaviours. However, the authors concluded that there was a deficiency in the implementation of a comprehensive dental examination before and after the program [24].

A four-week public-funded oral rehabilitation program, specifically known as the public-funded denture program, was introduced in a prospective pre-post comparison study conducted in India. The program primarily targeted edentulous senior citizens aged ≥ 60 years. Although the observed effectiveness in enhancing oral health quality of life among the edentulous elderly is worth acknowledging, the authors concluded that it would be significant to further investigate the favourable aspects of the natural settings, a significant sample size, and long-term reliability of the tools used to measure oral health-related quality of life (OHRQoL) before generalizing the findings to different contexts [15].

The prevalence of tooth loss among Black individuals in the USA has been noted. To understand whether a usual source of health care helps mitigate racial differences in complete tooth loss and recent dental visits among urban and rural older adults, Caldwell and colleagues evaluated the Primary Care for the Oral Health program. The target population consisted of adults aged ≥ 50 years. Access to primary health care was found to be associated with improved oral health outcomes, but the authors concluded that it did not completely close the gap between Whites and Blacks in rural communities. However, the inclusion of the US region and tract-level poverty improved the description of how living in a rural area may contribute to racial differences in oral health status. To comprehend variations in dental health in later stages of life as well as differences in fluoride exposure, the authors recommended that future research should explore the childhood experiences of rural White and Black adults [10].

### Category 2: School-based oral health community engagement interventions

Schools can play a significant role in promoting children’s oral health and overall wellbeing. By implementing school-based oral health programs, schools can extend the reach of oral health education, preventive measures, and services to children within school-age groups. Therefore, this category included articles that utilized such programs to offer guidance and support for oral health care. Two interventional studies were conducted on primary school children to evaluate a 6-month school-based program. Both studies showed that the program was effective in enhancing tooth brushing, knowledge, and attitudes, as well as in reducing the oral hygiene index-simplified, plaque index, and gingival index scores. These studies were conducted in Tanzania [1] and India [2] and implemented the Atraumatic Restorative Treatment (ART)/Oral Health Education (OHE) program and the Teacher-Dentist Dental Health Education (DHE) program, respectively. However, in the Tanzanian study [1], the short-term intervention program did not result in a decrease in sugar consumption, indicating that behavioural change is a gradual process that requires time, whereas an Indian study [2] indicated that the effectiveness of Dental Health Education was higher when conducted regularly by trained teachers than when conducted by qualified dentists. This suggests that after receiving brief training, teachers can also perform periodic screenings for visible build-up of plaque and calculus in children.

In contrast, another interventional study was conducted in India to implement and evaluate community participatory oral health promotion and prevention programs in school children as well as to conduct knowledge, attitude, and practice surveys among health workers and schoolteachers on oral health in a 16-month Participatory Oral Health Promotion and Prevention Program. The results of the study showed significant improvements in filled surface lesions of both the primary and permanent dentition, as well as in the bleeding sextants. The authors concluded that empowering the local community to provide oral health education and basic preventive treatment would bridge the oral health inequality gap between urban and rural communities, particularly among schoolteachers who can effectively impart oral health education to school children on a long-term basis [23].

Surprisingly, the 3-month program conducted in schools that focused on preventing tooth decay through teaching proper oral hygiene and supervised toothbrushing successfully increased oral health knowledge and cultivated positive oral hygiene practices among school children. However, the authors noted that despite being the shortest program, the most positive results were obtained when they included additional activities. These activities included assessing the nutritional status and hidden sugar in each child’s diet using a 3-day diet diary and conducting frequent oral examinations to motivate children to improve their brushing technique [3].

Despite significant technological advancements in dentistry, the lack of a sufficient dental workforce remains a significant obstacle for underprivileged communities to access dental and oral healthcare services. In 2016, the USA implemented the TeleHealth Network Grant Program in schools, in a study conducted by Ward *et al*. (2022). Despite the findings indicating that the method allowed traveling clinicians to successfully carry out almost all (97%) of the necessary treatment within their professional boundaries, it significantly decreased the necessity for a physical visit to a dentist (52%). The need for dental hygienists in schools and the use of telehealth technology to collaborate with dental professionals can improve access to oral healthcare services for children in rural communities. In addition, the authors suggested that dental hygienists could utilize teledentistry to perform oral health screenings in schools, thereby effectively identifying school children who have a higher risk of dental caries. This method may be beneficial in the prevention and management of oral diseases [20].

### Category 3: Integrated dental-based oral health community engagement interventions

Studies have shown that communities with access to oral health professionals experience improved oral health [40,41]. These professionals are trained to create personalized oral health plans and take preventive measures to enhance the oral health of communities [42]. Furthermore, their participation is crucial for improving referral pathways and implementing programs for oral health management [40]. Therefore, this category included articles that underpinned integrated dental-based oral health community engagement programs. For example, Dental assistants were trained to apply fluoride varnish to Aboriginal primary school children to mitigate disparities in access to oral healthcare services. Skinner *et al*. (2020) investigated the feasibility of using such personnel in a 12-month Aboriginal Dental Assistant Scholarship Program and a Fluoride Varnish Program. Their study found that dental assistants were effective in safely applying fluoride varnish to primary school children, with no reported adverse reactions. Additionally, no complaints were received from students, parents, guardians, schoolteachers, or principals regarding participation in or conduct of the study [14].

Similarly, in Australia, during the 3-year integrated partnership between the Oral Health Therapy Graduate Year Program and the Dalang Project, which promoted oral health service delivery and promotion, Skinner *et al*. (2021) investigated the interest of graduates working in rural communities following their participation in the collaborative project. Although the project was successful in improving oral health services for Aboriginal children and provided a positive experience for oral health therapists, many graduates continued to work in rural, remote, and regional locations after completing the program [16].

A community health center (CHC) in Washington, USA, introduced a medical-dental integration program (MDI) for children. The program allowed medical and dental providers to examine children during the same visit at a paediatric medical clinic or women, infants, and children program location in a rural community. The main aim of the program was to increase access to oral health care and to mitigate childhood caries. Consequently, Pawloski *et al*. (2022) evaluated the acceptability, feasibility, and success of an MDI integration strategy in a CHC setting and determined that it was acceptable and feasible owing to the collaborative approach that included the involvement of service providers, leadership alignment, and support, consistent and clear communication, and employment of a registered dental hygienist as the oral health provider [19].

In Australia, the Kimberley Dental Team (KDT) was founded as a non-profit and volunteer organization in 2009. Their objective was to deliver dental care and education to Aboriginal children and their families residing in the Kimberley area. Despite the initiative being in place for a long period, the Aboriginal population in the Kimberley region continues to face challenges in obtaining oral health services, experiencing inadequate nutrition, and a lack of government assistance. These obstacles persist despite the advantages of education and access to appropriate culturally sensitive care, which were previously believed to contribute to better oral health outcomes [5].

### Category 4: Non-dental-based oral health community engagement interventions

Proper allocation of skilled and motivated healthcare workers in a timely and suitable manner is crucial for ensuring efficient healthcare services and improving health outcomes [43]. While non-dental personnel can provide temporary relief from symptoms and refer individuals to oral health professionals [44], their involvement in communities can result in the sharing of oral health knowledge and influence decision-making [45]. As a result, this scoping review included articles on oral health programs that guide oral health care through non-dental personnel. For instance, the study conducted by Fernando *et al*. (2015) demonstrated that the enhancement in the oral health of preschool children was statistically significant and was attributed to the intervention aimed at educating mothers and caregivers of preschool children. This finding suggests that non-dental personnel can effectively deliver oral health education to improve the oral health of children. This experimental study was conducted in Sri Lanka to evaluate a 6-month Non-Dental Personnel Program [4].

Similarly, regarding the community health worker program, Erchick *et al*. (2020) conducted a prospective cohort study that evaluated the validity of periodontal examinations conducted by auxiliary nurse midwives in a rural home setting in Nepal. Although the study found that the overestimation was minor and unlikely to have an impact on population-based estimates of important indicators of oral health status, it was recommended that certain basic oral health services be transferred from dentists and other highly trained professionals to auxiliary nurse midwives or community health workers. These conclusions were drawn after auxiliary nurse midwives tended to report higher periodontal probing depth scores relative to dentists [11].

In the WHO health-promoting school framework in Uganda, Akera *et al*. (2022) concluded that oral health promotion in primary school children requires the support of teachers, parents, health workers, and community leaders. Regular training is necessary for teachers to improve their skills and to provide dental services. Local, district, and national resources are necessary to support oral health promotion in school children [17].

## Discussion

The purpose of this scoping review was to identify oral health community engagement programs that guide oral health care in rural communities and to provide an overview of their outcomes. To achieve this, we employed a systematic approach to identify records bearing oral health community engagement programs, which were then categorized into four main intervention groups: community-based, school-based, integrated dental-based, and non-dental volunteer-based strategies. The eligible records that we obtained originated from both developed [15,25,27,30–34] and developing countries [16–18,22–24,26,28,29,35,36]. Overall, the results of this scoping review confirmed that oral health community engagement programs were effective in enhancing the knowledge and perception of oral health among children and mothers/caregivers; improving oral health and quality of life of edentulous older adults and children; expanding access to and incorporating oral healthcare services provided by dental personnel; and improving oral health outcomes through the involvement of community health workers and teachers among rural residents. Despite these achievements, the current scoping review highlighted a persistent and significant gap in the literature regarding the implementation, evaluation, and impact of oral health community engagement programs in rural communities. This gap pertains to individuals’ need for shared responsibilities and an understanding of oral health community engagement programs as well as their content.

It is important to highlight that there was a scarcity of literature available on oral health community engagement programs in this scoping review. While the majority of the records included in this scoping review were quantitative studies, mixed-method, and qualitative studies were also included. This observation may indicate a growing recognition of the value of qualitative data in enhancing oral healthcare interventions and outcomes [46], especially concerning oral health community engagement programs in rural communities.

Although the overall findings of this scoping review revealed oral healthcare improvements in various rural communities, the findings also suggested that developing countries face a substantial burden of oral diseases [16–18, 22–24,26,28,29,35,36]. Furthermore, the inclusion of studies evaluating oral health strategies in rural communities of developed countries provided evidence of persistent discrepancies in oral healthcare accessibility and a shortage of oral healthcare professionals, confirming that this burden is of global significance [15,25,27,30–34]. The results of the scoping review also indicated that the methods used to evaluate the effectiveness of the oral health community engagement programs implemented in rural communities of both developing and developed countries were inconsistent (Table 2). Therefore, these findings were inconclusive, as it was discovered that all the intervention studies were only conducted in rural communities of developing countries and had used a variety of methods to measure or compare the effectiveness of oral health strategies [16–18,23,24,28,29], but none of the intervention studies were conducted in developed countries. These findings are supported by the report, which suggested that in developing countries, oral health services are predominantly focused on the delivery of emergency care and targeted interventions within the various populations [47].

The current study also found that, out of the 11 oral health community engagement programs implemented to guide oral health care in rural communities of developing countries, Dental Health Education was the only strategy implemented in such settings [22]. Furthermore, in Africa, only two oral health strategies have been implemented and evaluated, both of which were implemented in the eastern part of the continent and were focused on promoting oral health in primary schools [18,36]. These findings suggest a significant inadequacy and deficiency in the provision of oral health education in developing countries. Similarly, the majority of oral health community engagement programs were implemented in Asia [16,17,23,24, 26,28,29,35], where the continent stands out as the only one that has successfully implemented a diverse range of oral health strategies to guide oral health care for various populations, such as children, mothers/caregivers, pregnant women, edentulous individuals, teachers, and geriatric patients. However, the implementation of these strategies may be driven by the objective of mitigating the complex issue of approximately 900 million cases of untreated dental caries, severe periodontal disease, and edentulism prevalent throughout the continent [48].

In contrast, the results of this scoping review revealed that integrated dental-based oral health community engagement programs were only implemented in rural communities of developed countries and were successful in expanding access to and incorporating oral healthcare services provided by dental personnel [25,28,32,35]. Although disparities in access to oral health care have been demonstrated to be universal challenges, rural communities in developed countries have better access to oral healthcare services than those in developing countries. These findings may be attributed to the availability of a variety of dental personnel with different skill mixes integrated into oral health strategies to provide oral health services in these settings, such as dental assistants, oral health therapist graduates, dentists, and medical practitioners [25,28,32,35]. This finding further confirms a significant shortage of available and effective oral health community engagement programs that consist of integrated dental personnel to provide oral healthcare services in rural communities in developing countries.

This scoping review also revealed that diverse oral health community engagement programs, specifically aimed at improving the oral health of children in primary schools, were successful, as most children showed improved toothbrushing techniques, improved oral health knowledge, positive attitudes, and reduced prevalence of dental caries [15–18,22,28–30,36]. Although the teachers were integrated and able to successfully incorporate oral health promotion into primary school oral health programs, various obstacles may hinder the long-term viability of this approach. These obstacles may include limited time, excessive workloads, the absence of an accountable person for the program, food stalls and vendors, gaps in the curriculum, lack of cooperation, and scarce resources [49]. Furthermore, the integration of the TeleHealth Network Grant Program in a rural school in a developed country proved successful in bridging the gap between the rural community’s primary school children and access to oral health care [36]. Nevertheless, the likelihood of this technology being successful in rural communities may be limited due to the significant influence of poor infrastructure and inadequate services such as electricity and telecommunications on the provision of services [43].

In response to the WHO’s recommendations to improve access to healthcare workers in rural communities [43], three community engagement programs for oral health were implemented with a focus on non-dental personnel to provide oral health services in rural communities. This scoping review found that deploying non-dental personnel was an economically viable strategy, as it leads to significant cost savings by utilizing local resources. However, concerns have been raised, and they include poor sustainability of oral health strategies and the lack of government support for resource allocation for oral health promotion and continuous training for oral health education among these personnel [16,35,36]. Furthermore, improving the attraction, recruitment, and retention of the workforce regardless of their level of economic development is part of the WHO’s strategies to increase the number of community healthcare workers in rural communities [43]. However, according to the results of this scoping review, no community healthcare workers were integrated into any of the oral health community engagement programs implemented in the rural communities of developed countries to guide oral health care (Table 2). This finding may be in accordance with the finding that healthcare facilities in developed countries are potentially furnished with cutting-edge technical equipment and reinforced by sufficient oral health professionals specializing in diverse domains, thereby facilitating collaborative efforts aimed at ensuring optimal patient outcomes [47].

The current comprehensive scoping review yielded substantial evidence demonstrating the effectiveness of community-based oral health programs in guiding and enhancing the knowledge and perception of oral health among children and mothers/caregivers and improving oral health and quality of life of children and edentulous older adults (Table 3) [15,23–27]. Although the objective of these programs was to enhance access to oral healthcare services for all rural communities in developed and developing countries, the general members (patients) of these communities were given lower priority and only received limited benefits from these programs. That is, of the seven community-based programs implemented, only one oral health strategy was designed to address the oral health needs of general members (patients) [31]. Based on this finding, it is evident that existing oral health strategies only target specific populations and neglect the general public.

### Strengths and limitations

To the best of our knowledge, this is the first comprehensive scoping review to identify and provide detailed information on community-based oral health promotion and prevention programs/strategies in rural communities. We ensured the rigorous application of eligibility criteria by including only peer-reviewed articles. This review captured the perspectives of various stakeholders, such as schoolteachers, parents/caregivers, children, community health workers, and volunteers. After consulting with a health science librarian, we conducted a thorough search of seven databases and chased citations of previously published articles and eligible studies without any restrictions on publication type or region, which ensured the capture of all relevant literature and minimized the risk of selection bias. The limitations of this scoping review are grounded in the inclusion and exclusion criteria. In particular, the criterion that all records be written in English may have introduced bias by excluding relevant records in other languages. In addition, other materials, such as abstracts, dissertations, and white papers, which may have provided relevant information, were omitted in this scoping review. The interpretation of the concept of the "rural community" is inconsistent and can be expressed in various ways. Consequently, some records obtained may relate to the concept but were not labelled as such by the authors. Therefore, these records were not included in the final analysis of the scoping review.

### Recommendations

This scoping review suggests that it is advisable for oral health community engagement programs, both in the present and in the future, to prioritize integrated dental-based strategies. This approach should also include the use of Dental Assistants to ensure the optimization of oral health education-based strategies. By doing so, the emphasis would shift away from curative-oriented strategies and instead be inclusive of all rural residents rather than exclusively targeting specific populations.

## Conclusion

This scoping review describes oral health community engagement programs for guiding oral health care for rural communities. The results suggest that there is an increasing understanding of the importance of qualitative data in improving oral healthcare interventions and outcomes. The results also indicate that oral health strategies were effective in guiding the knowledge and perception of oral health among children and mothers/caregivers and in improving the oral health and quality of life of children and edentulous older adults in rural communities. However, rural communities are still heavily burdened by oral diseases, owing to unequal access to dental care and a shortage of oral health professionals. The existing oral health community engagement programs for engaging with these communities have used inconsistent methods to assess their effectiveness. This study also found a significant gap in the provision of oral healthcare services in these communities, owing to the absence of programs that integrated dental personnel as well as a lack of sustainability and government support for resources to promote oral health.

## Supporting information

S1 Table Search strategy. (DOCX)

S2 Table PRISMA scoping review checklist. (DOCX)
