# Supplementary material for: Oral health community engagement programs for rural communities: A scoping review
Source: PLoS One. 2024 Feb 6;19(2):e0297546. doi: 10.1371/journal.pone.0297546 (PMC10846741; doi:10.1371/journal.pone.0297546)
Supplement: S1 Table — (DOCX) [file pone.0297546.s001.docx]

**S1 Table. Search strategy**

| Database | Search combination terms | Limit |
| --- | --- | --- |
| PubMed | (("mouth"[MeSH Terms] OR "mouth"[All Fields] OR "oral"[All Fields]) OR ("dental health services"[MeSH Terms] OR ("dental"[All Fields] AND "health"[All Fields] AND "services"[All Fields]) OR "dental health services"[All Fields] OR "dental"[All Fields])) AND ("health"[MeSH Terms] OR "health"[All Fields]) AND (rural[All Fields] OR remote[All Fields]) AND rural[All Fields] AND (("residence characteristics"[MeSH Terms] OR ("residence"[All Fields] AND "characteristics"[All Fields]) OR "residence characteristics"[All Fields] OR "communities"[All Fields]) OR settings[All Fields] OR areas[All Fields]) AND (("residence characteristics"[MeSH Terms] OR ("residence"[All Fields] AND "characteristics"[All Fields]) OR "residence characteristics"[All Fields] OR "community"[All Fields]) AND ("social participation"[MeSH Terms] OR ("social"[All Fields] AND "participation"[All Fields]) OR "social participation"[All Fields] OR "engagement"[All Fields]) OR participation[All Fields] OR outreach[All Fields] OR programs[All Fields]) AND (frameworks[All Fields] OR ("guideline"[All Fields] OR "guidelines as topic"[MeSH Terms] OR "guidelines"[All Fields]) OR strategies[All Fields]) | English  2012 - 2023 |
| Scopus | TITLE-ABS-KEY ( ( oral OR dental ) AND ( health ) AND ( rural OR remote ) AND ( rural ) AND ( communities OR settings OR areas ) AND ( community AND engagement OR participation OR outreach OR programs ) AND ( frameworks OR measures OR guidelines OR strategies ) ) | English  2012 - 2023 |
| EBSCOhost | "( oral OR dental ) AND ( health ) AND ( rural OR remote ) AND ( rural ) AND ( communities OR settings OR areas ) AND ( community AND engagement OR participation OR outreach OR programs ) AND ( frameworks OR measures OR guidelines OR strategies ) ) | English  2012 - 2023 |
| Wiley Online | [All: oral] AND [All: health] AND [All: rural] AND [[All: communities] OR [All: rural]] AND [[All: settings] OR [All: remote]] AND [All: areas] AND [All: community] AND [[All: engagement] OR [All: community]] AND [[All: participation] OR [All: community]] AND [[All: outreach] OR [All: outreach]] AND [[[All: programs] AND [All: frameworks]] OR [All: conceptual]] AND [[All: framework] OR [All: strategies] OR [All: guidelines] OR [All: measures]] | English  Jan 2012 - 2023 |
